# Xiaoqinglong Granules as Add-On Therapy for Asthma: Latent Class Analysis of Symptom Predictors of Response

**DOI:** 10.1155/2013/759476

**Published:** 2013-02-03

**Authors:** Qinglin Zha, Seqi Lin, Chi Zhang, Christopher Chang, Hanrong Xue, Cheng Lu, Miao Jiang, Yan Liu, Zuke Xiao, Weiyou Liu, Yunfei Shang, Jianjian Chen, Minyong Wen, Aiping Lu

**Affiliations:** ^1^School of Computer, Jiangxi University of Traditional Chinese Medicine, Nanchang 330004, China; ^2^Institute of Basic Research in Clinical Medicine, China Academy of Chinese Medical Sciences, Beijing 100700, China; ^3^Division of Allergy and Immunology, Thomas Jefferson University, Wilmington, DE 19803, USA; ^4^Department of Respiratory Medicine, Affiliated Hospital of Jiangxi University of TCM, Nanchang 330004, China; ^5^Department of Respiratory Medicine, People's Hospital of Jiangxi Province, Nanchang 330006, China; ^6^Department of Respiratory Medicine, The First Affiliated Hospital of Gannan Medical University, Gannan 314000, China; ^7^Department of Respiratory Medicine, The Third Affiliated Hospital of Nantong University, Nantong 216000, China; ^8^Department of Respiratory Medicine, Nanchang Hospital of Integrated Traditional Chinese and Western Medicine, Nanchang 330003, China; ^9^Department of Respiratory Medicine, The First Affiliated Hospital of Guangzhou University of TCM, Guangzhou 510405, China; ^10^School of Chinese Medicine, Hong Kong Baptist University, Kowloon Tong, Kowloon, Hong Kong

## Abstract

Xiaoqinglong granules (XQLG) has been shown to be an effective therapy in asthma animal models. We reviewed the literature and conducted this study to assess the impact of XQLG as an add-on therapy to treatment with fluticasone/salmeterol (seretide) in adult patients with mild-to-moderate, persistent asthma. A total of 178 patients were randomly assigned to receive XQLG and seretide or seretide plus placebo for 90 days. Asthma control was assessed by asthma control test (ACT), symptoms scores, FEV_1_, and PEF. Baseline patient-reported Chinese medicine (CM)-specific symptoms were analyzed to determine whether the symptoms may be possible indicators of treatment response by conducting latent class analysis (LCA). There was no statistically significant difference in ACT score between two groups. In the subset of 70 patients with symptoms defined by CM criteria, XQLG add-on therapy was found to significantly increase the levels of asthma control according to global initiative for asthma (GINA) guidelines (*P* = 0.0329). There was no significant difference in another subset of 100 patients with relatively low levels of the above-mentioned symptoms (*P* = 0.1291). Results of LCA suggest that patients with the six typical symptoms defined in CM may benefit from XQLG.

## 1. Introduction

Complementary or alternative medicine (CAM) is extensively used in the treatment of asthma. In spite of the multitude of Western medicine treatments available for patients with asthma [1–4], only 41% of UK asthma sufferers have not used CAM and of those two-thirds would consider using it in the future [5]. Recommendations for the use of CAMs should be based only on rigorous proof of efficacy derived from high-quality studies [6]. Chinese herbal medicines (CHMs) are a consistently popular form of CAMs in asthma and proprietary asthma drugs are derived from herbal remedies [7]. Some of these herbal remedies have a millenary history and represent the traditional medicine in many countries. Xiaoqinglong decoction (XQLD), also known as Sho-seiryu-to (SST) in Japan and So-cheong-ryong-tang (SCRT) in Korea, was first written about by Zhang Zhong Jing, in the Traditional Chinese Medicine (TCM) classic text Shang Han Lun (Treatise on Cold Damage Diseases), about 1,800 years ago [8, 9]. There are eight herb components in the medicine. These constituents and their actions are described in Table 1 [10, 11].

XQLD is a popular intervention which has been used for the treatment of asthma triggered by upper respiratory infections [13]. It is most suitable when phlegm is excessive, clear, and watery and the body feels chilly [14, 15]. Accordingly, XQLD is not used to treat dry cough or cough with thick or yellow phlegm [11]. Since TCM was brought to Japan and many other Asian countries [16], XQLD has had a very great influence on the practice of medicine. A large sample-sized retrospective study indicates that XQLD is a very commonly used formula in Taiwan [8, 17]. XQLD has attracted Asian scientists with an interest in investigating mechanisms of action since the 1980s, particularly in Taiwan [18] and Japan [19].

### 1.1. A Review of the Pharmacologic and Clinical Effects of XQLD

The general pharmacological properties of XQLD extracts were investigated in various experimental animals [20]. XQLD has been reported to show antiallergic effects [21–23] and appears to be useful for treating type I allergic reactions. There are few antihistaminic side effects, such as the sedation and drowsiness that result from blockade of histamine H1 and muscarinic receptors in the brain [24]. The suppressive activity by XQLD on chemical mediators, such as histamine and leukotrienes from peritoneal mast cells in vitro, has also been demonstrated [23].

A previous study in guinea pigs demonstrated that the antiasthmatic effects of XQLD appeared to be partly mediated by stimulation of *β*2-adrenoceptors, leading to broncho relaxation, and that XQLD inhibits the infiltration of eosinophils into the airway [25]. Fresh data on asthma has shown that at least a part of the mechanism of action of XQLD against OVA-sensitized allergic airway inflammation in a mouse model is different from that of prednisolone, and the results demonstrate that XQLD alleviates nasal symptoms by the inhibition of histamine signaling through suppression of TDI-induced H1R and HDC gene upregulation [19].

In addition, due to the multiple components in XQLD, there may be other mechanisms of action. For example, XQLD appears to inhibit mast cell degranulation [26] and influence CD4(+) T cells, which may be significant in developing its use in the treatment of IgE-mediated allergic asthma [16]. The role that XQLD may play in the regulation of cytokines and chemokines and the subsequent effect on allergic airway inflammation is not known [27]. Kao and colleagues demonstrated that XQLT exhibits antiairway inflammatory, anti-airway remodeling, and specific immunoregulatory effects in a chronic asthmatic mice model [28]. Their results indicated that XQLD treatment alleviates asthma-like pulmonary inflammation via suppression of specific chemokines [29]. Since many physicians frequently prescribe XQLD in combination with conventional Western medicine, researchers evaluated the pharmacokinetic interactions in rats to evaluate the potential for adverse effects of the combined therapy [30, 31].

Similarly, according an experimental study from Japan, authors examined whether XQLD administration is affected by a probiotic product and concluded that XQLD-mediated anti-allergic effects are enhanced by the probiotic [32]. The XQLD constituent herbs, including Herba Ephedrae and Herba Asari, have been reported to have cytokine-modulating effects [33]. Accordingly, XQLD has been used not only for “symptomatic” treatment of allergic diseases but also for “prevention.” It is important to note that the pharmacokinetics of the ephedrine component of XQLD has also been investigated [34, 35].

Previous clinical trials of XQLD were nonrandomized or randomized to include a standard of care control group. In these initial studies, XQLD was associated with a decrease in the long-term rate of mild asthma exacerbations, improvement in asthma control, and improvements in lung function [13]. A previous clinical trial has also demonstrated its potential benefits in improving quality of life in asthmatics [36]. Unfortunately, most of the previous clinical trials on XQLD are of poor methodological quality and therefore XQLD remains of uncertain value in the treatment of asthma [13]. Since XQLD remains popular for the treatment of asthma in Asian communities, this study was conducted to further elucidate the efficacy and safety of XQLD in the treatment of asthma.

### 1.2. The Current Status of XQLD Research in Asthma

To our knowledge, this is the largest randomized positive-controlled trial to test XQLD in the treatment of asthma. Trial designers were concerned about standardized intervention because many of TCM decoctions cannot ensure reproducibility of therapeutic effects. Xiaoqinglong granules (XQLG), a Chinese patent medicine of the same name as XQLD, has the same proportions of ingredients and is manufactured in accordance with the Chinese Pharmacopoeia [12]. In Japan, most uses of granules are based upon classical formulas. The vast majority of the formulas from the Shang Han Lun are particularly prominent in modern clinical use. Granules have also become deeply integrated into healthcare in Taiwan over the past 30 years [37]. This development was largely stimulated by the convenience of granules for patients, along with the fact that the governments of both Japan and Taiwan also streamlining the process for applications exclusively when granules products apply for classical formulas coverage [38]. This has spurred a tremendous amount of growth and economic investment, as well as widespread clinical use and the experience that comes with increased use. Granules are desirable for large scale healthcare distribution because they are well regulated and hygienic, and they are ideal for research because batches are consistent and traceable.

Likewise, the Chinese government maintains the registration administration mechanism that oversees TCM combination preparations originating from historic, classic, and well-known recipes. The application data for marketing TCM combination preparations may be partially exempted based on formulation and source as well as their composition, function, indications, and manufacturing procedures [39]. However, perhaps as a result of this streamlining of the process for phase I, II, and III trials, there are certain barriers to obtaining more specific indications and detailed safety assessment reports of current products. High quality research is needed in the area of efficacy and safety of traditional Chinese herbal medications.

### 1.3. Chinese Medicine Patterns of Asthma

The aim of the current study, therefore, is precisely to determine the effectiveness and safety of XQLG for the treatment of asthma in adults. In addition, the predictive value of the baseline characteristics with respect to response to the therapy is investigated due to the importance of clinical characteristics, as reported in previous studies. Similarly, Chinese medicine (CM) symptoms are very important for CM diagnostics in daily clinical practice. In CM, a specific set of symptoms is referred to as a pattern. CM pattern classification has been used to define subsets of patients within a few thousand years. This is analogous to the development of personalized medicine in Western medicine concepts, where active research is being conducted to define genetic or nongenetic characteristics that predict treatment response to new or existing drugs. The additional specific study aims are therefore to (1) use latent class analysis, a statistical model to classify asthma adults with symptoms predicators; (2) provide evidence of latent class concurrent validity by examining association between class membership and unmet healthcare needs.

## 2. Methods

### 2.1. Patients

Male and female patients (aged 18–65 year) with a documented history of asthma as defined by the National Institute of Health (NIH) [40] and Global Initiative for Asthma (GINA) [41] were eligible for study entry. Other inclusion criteria were reversibility of baseline forced expiratory volume in one second (FEV_1_) (≥15% and ≥200 mL) in response to inhaled salbutamol, and “asthma of cold pattern” according to Criteria of Diagnosis and Therapeutic Effect of Disease and Syndromes in Traditional Chinese Medicine [42]. According to the guidelines of asthma treatment and prevention in China (GATPC) [43], patients are divided into three types, including acute exacerbation, chronic persistent, and clinical remission. This trial included only participants with mild-to-moderate persistent asthma.

Exclusion criteria included the following. Contraindicated medications included theophylline, corticosteroids, and short-acting beta2 agonist (SABA) therapy within the past two weeks; any significant disease or disorder that may influence results or a patient's ability to participate in the study; “asthma of heat pattern” according to Criteria of Diagnosis and Therapeutic Effect of Disease and Syndromes in Traditional Chinese Medicine [42]; women who were pregnant or breastfeeding; known hypersensitivity to any intervention; participation in other investigational studies in the last 4 weeks.

The study was conducted in accordance with the Declaration of Helsinki and was approved by local ethics committees and institutional review boards as appropriate. All patients provided written informed consent prior to participating in the study.

### 2.2. Study Design

**Figure 1:**
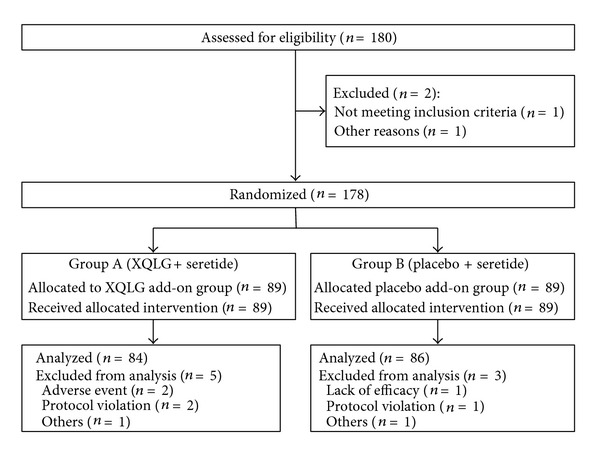
Flow Diagram of the enrollment progress through the phases of XQLG randomized trial of the two groups.

A randomized, double-blind, positive controlled, parallel-group, multicenter study was conducted at the six centers in China. The study, focusing on a superiority study design, was sequentially conducted as follows: a run-in period of one week prior to randomization, followed by a treatment period of 90 days. At the end of the run-in period, participants were randomized to the active XQLG/seretide group (Group A) or placebo/seretide group (Group B) by the central randomization system (Figure 1). This system is provided by China Academy of Chinese Medical Sciences, which adopted the computer telephone integration (CTI) technology to integrate computers, internet, and telecom. The random number list is assigned by interactive voice response (IVR) and interactive web response (IWR) [44]. The success of blinding will be assessed at each participant's last visit. An assessor who did not participate in the treatment and who is blinded to the allocation results will perform the outcome assessment.

Sample size calculation was projected with the use of SAS software. Based on our pilot trial, the response rate after 90 days was estimated at 75% in the conventional treatment group and 94% in XQLG add-on therapy group (level of significance 5%, power of 90%).

## 3. Treatment

### 3.1. XQLG

 Proprietary name: Xiaoqinglong granules.
 Chinese pronunciation: Xiaoqinglong Keli.

Xiaoqinglong granules (State Food and Drug Administration (China) Approval no. Z19993076) used was manufactured by Beijing Shouer Pharmaceutical Factory (Beijing, China) (batch no. 091201).

Ingredients: Herb Ephedrae, Ramulus Cinnamomi, Rhizoma Zingiberis, Radix Asari, Fructus Schisandrae, and Radix Paeoniae Alba (Table 1).

Quality control: Beijing Shouer Pharmaceutical Factory is a GMP certificated pharmaceutical factory. Its product (batch no. 091201) complies with the requirements of Chinese Pharmacopoeia 2005. According to pharmacopeia requirement, compounds Ephedrine hydrochloride, Pseudoephedrine hydrochloride, and Paeoniflorin in Xiaoqinglong granules sample were determined (6.232 mg/g, 2.853 mg/g, and 0.8538 mg/g, resp.) and fully comply with Chinese pharmacopeia 2005 standard. Identification details refer to Chinese Pharmacopoeia (2005 edition).

Description: light brown to brown granule, or grey to light brown, smells slightly fragrant, tastes sweet and slightly pungent.

### 3.2. Seretide

Seretide is a combination medication containing an inhaled corticosteroid (fluticasone) and a long acting reliever or symptom controller (salmeterol). This combination of salmeterol xinafoate and fluticasone propionate is recommended by GINA as a regular treatment for uncontrolled asthma [41].

Subjects in the XQLG add-on group received oral XQLG (taken after being dissolved in boiled water, 13 grams/1 pack, three times a day) and seretide (1 puffs twice daily). Subjects in the seretide group received xiaoqinglong placebo granules for 90 days and seretide (1 puffs twice daily).

Subjects requiring additional intervention at any time because of disease severity were withdrawn from the study. For the duration of the study, inhaled Ventolin (100 to 200 *μ*g) 3 to 4 times daily, not exceeding 8 puffs (800 *μ*g/day), was allowed as needed.

### 3.3. Clinical and Laboratory Evaluation

The primary outcomes were the achievement of asthma control and asthma control evolution. Asthma control achievement was based on clinical control (as recommended by GINA 2006) [41, 45]. Asthma control evolution was measured with the ACT (Dutch version). The ACT is a clinically validated measure for asthma control, consisting of five questions, each having five possible response modalities (classified by decreasing levels of asthma control, scored from 5 to 1) [46, 47]. The ACT score (range 5–25) was determined by summing the response scores to the five questions; the higher the score, the better the asthma control. The ACT was filled out by patients at the start of the run-in phase, at randomization and 7, 30, 60, and 90 days after randomization.

Secondary outcomes included changes in lung function pre- and posttests (FEV_1_, PEF). Asthma symptom scores, measured on a scale of 0 to 6 (0 = no symptoms; 2 = mild symptoms; 4 = moderate symptoms; 6 = severe symptoms), were also recorded. These scores were summed to obtain the total daily asthma symptom score in 7, 30, 60, 90 days. Safety evaluations included incidence of treatment-emergent adverse events (AEs) and serious adverse events (SAEs).

### 3.4. Statistical Analysis

All randomized subjects were assessed by comparing baseline characteristics of both study groups using unpaired *t*-tests for continuous variables, and chi-squared tests or Wilcoxon rank sum test for categorical variables. The primary analysis was performed on the intent-to-treat (ITT) approach including all randomized patients with a baseline value and at least 1 treatment period measurement. The ACT scores measured at baseline and 90 days were analyzed using a repeated measures multivariate ANOVA. Asthma control (GINA 2006) was analyzed using Wilcoxon rank sum test at endpoint. Missing data for secondary endpoints were imputed using the Last Observation Carried Forward method. Unless otherwise stated, results are reported as mean ± SD. Assumptions of normality and homoscedasticity were assessed. All statistical tests were 2 tailed, and *P* values less than .05 were considered statistically significant. The safety evaluation included all randomized patients. The number and percentage of patients reporting clinical adverse experiences were summarized by treatment group.

Given the identical protocols and measurements, baseline data for Group A (Figure 1) and Group B clinical studies were combined for the subset analyses. The latent classes were determined based on presence or absence of 8 symptoms: Qi vacuity, chest tightness, aversion to cold, clear, pale, or watery phlegm, torpid intake, coughing, fever, and no thirst.  Table 2 presents each of the variables and how they were categorized.

In order to examine the structure underlying the set of 8 symptoms, latent class analysis (LCA) was performed using SAS PROC LCA (SAS version 9.2, Cary, NC, USA: SAS Institute Inc.) [48]. LCA is a statistical method that is used to classify subtypes of patients according to selected TCM clinical characteristics and estimate the appropriate number of latent classes based on the lower Bayesian information criteria (BIC) value and the Akaike information criterion (AIC). In PROC LCA, missing data are managed by maximum likelihood using an expectation-maximization (EM) procedure, with data assumed to be missing at random.

## 4. Results

A total of 178 patients underwent randomization, of which 170 patients comprised the ITT population. Reasons for patient withdrawal at the screening and randomization stages are summarized in Figure 1. Nearly half of the patients were men (47.6%), and the age ranged from 19 to 65 year. Baseline demographics and clinical characteristics were well matched between groups (Table 3). The subjects enrolled in this trial had inadequately controlled asthma as defined by the inclusion criteria of the study design and a low ACQ score consistent with poorly controlled asthma.

### 4.1. Asthma Outcomes

Compared with baseline, the improvements in uncontrolled levels of asthma control according to GINA guidelines during the 90-day treatment period were observed both in the XQLG 13 g three times daily combination therapy and control groups (uncontrolled (%) from 90.48 to 6.58, from 93.02 to 11.25, resp.). However, no statistically significant difference between treatment groups was apparent (*P* values 0.8883) (Table 4), whilst significant amelioration of the predefined scores was seen in both groups. Similarly, although all treatment groups demonstrated a mean improvement in ACT score and asthma symptoms (Table 5), between-treatment differences were also not statistically significant. Estimated mean differences were 0.35 (95% CI, −1.49 to 2.19; *P* = 0.5334) and −1.13 (95% CI, −3.83 to 1.58; *P* = 0.1728), respectively, for 90-day evaluation of ACT Score and Asthma Symptoms. Overall, the improvements in ACT score and asthma symptoms with XQLG were paralleled by significant improvements in control group scores for 7 days, 30 days, 60 days, and 90 days (all *P* > 0.05).

**Figure 2:**
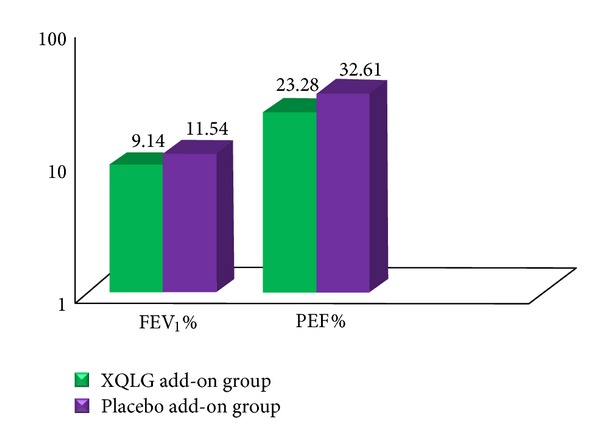
Adjusted mean change from baseline in forced expiratory volume (FEV_1_%) and peak expiratory flow (PEF%) during the entire 90-day treatment period.

Throughout the study period, the mean change from baseline in FEV_1_% increased for all treatment groups; at the end of the 90-day treatment period, FEV_1_% increased to 9.14% in XQLG add-on therapy and 11.54% for seretide. There was no statistically significant difference between the groups (Figure 2). The mean improvement in PEF% showed no between-treatment differences between the XQLG and control groups.

**Figure 3:**
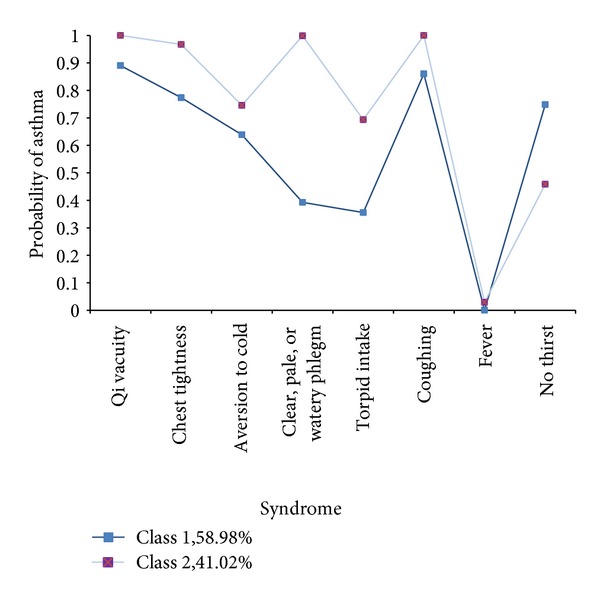
Conditional probabilities for each item endorsement according to latent class for asthma participants in subjects (*n* = 170).

LCA grouped all individuals into 2 classes (groups) according their symptoms. To determine the presence of latent classes for symptoms, 1 and 2 class models were tested. As shown in Table 2, item-response probabilities >0.5 in bold were defined to facilitate interpretation in a 2-class model. Class 1 consisted of 58.98% (*n* = 100) of the sample. As can be seen in Figure 3, the symptoms trend of adherence was similar in both classes. These participants were consistent in their responses to cold pattern symptoms. Class 2 consisted of the remainder 41.02% (*n* = 70) of the sample. Compared with participants assigned to class 1, class 2 participants had a higher probability of reporting adherence to the six typical symptoms, including Qi vacuity; chest tightness; aversion to cold; clear, pale, or watery phlegm; torpid intake; coughing. Conditional probabilities are provided in Table 2 and represented in Figure 3. Analyses were conducted to compare characteristics of class 1 to class 2 participants (Table 6). Duration of disease, age, gender, FEV_1_ (%), or PEF (%) did not appear to impact class membership. Even though there was a trend for amelioration of FEV_1_ in the XQLG add-on group, no significant differences were found. In the subset of 70 patients with the above-mentioned typical symptoms, XQLG add-on therapy was found to significantly increase the levels of asthma control according to GINA guidelines (*P* = 0.0329) (Table 7). There was no significant difference in the increase in the levels of asthma control between Groups A and B in the remaining subset of 100 patients with relatively low sensitivity of the above-mentioned symptoms (*P* = 0.1291) (Table 7).

### 4.2. Safety

Overall, treatment with XQLG and seretide was well tolerated. Neither group showed abnormal findings in hematology, or serum chemistry tests. No serious adverse effects were observed in either group. In particular, there were no deaths, hospitalizations, or emergency room visits. A total of one AE was reported by one patient (0.9%) in the XQLG add-on group with mild sinus tachycardia, which was of mild intensity, did not necessitate treatment discontinuation, and was not deemed to be treatment related by the investigator.

## 5. Discussion

The last decade has seen an increased interest in the use of TCM for asthma treatment, and effects have been documented in numerous conditions. Since 2005, several controlled clinical studies of “antiasthma” herbal remedies have been published in SCI indexed journals [49, 50]. XQLD has played an important role in preventing and treating lung diseases in China and other Asian countries for centuries where it is still used as a monotherapy or in integrated medicine. Such a wide spectrum of clinical efficacy suggests that the power of XQLG add-on therapy may help restore lung function. However, well-controlled clinical trials using herbs for the treatment of asthma are rare.

Using our randomized, double-blind, positive controlled, parallel-group design, we demonstrate that XQLG add-on therapy to seretide can significantly increase baseline levels of asthma control according to GINA guidelines ACT score, asthma symptoms score, FEV_1_, and PEF in asthmatic subjects. However, there were no differences between XQLG and control groups at the 90-days assessment. These findings are not completely consistent with the results of previous trials [13]. According to the published literature, some trials have identified significant differences in responses between XQLD add-on therapy and conventional western medicines although both of them were obviously better than baseline. Additionally, FEV_1_ and PEF were significantly better than conventional western medicines groups. In contrast, the data we obtained in the current study showed no clinically significant difference in the above parameters when XQLG was added to seretide, compared to when placebo was added. Thus it is necessary to include precise traditional criteria into the inclusion criteria of clinical studies on traditional herbal combinations.

The associations observed in this study must be viewed with a number of considerations. A possible interpretation of no clinically significant differences for the overall control of asthma may not take into account that certain specific characteristics of asthma control may in fact show clinically significant improvements, some of which may not be primary endpoints and may impact quality of life, such as days where normal activity is limited due to asthma symptoms, or visits to the emergency department or hospital. Some of these are included in the evaluation of a total asthma control symptom score, but these scores are frequently not broken down in clinical trials. Moreover, analysis of the entire group as a whole may not take into consideration that some patients with certain symptom patterns may in fact show a higher level of improvement if analyzed separately. A larger study will allow for stratification of patients into different groups based on age, socioeconomic status, gender, and so forth. In our case, screening patients with symptoms that CM identifies as important in asthma status may help to identify response to specific treatments in specific groups. The magnitude of pattern effect in a TCM clinical trial is often calculated as the benefit in the TCM arm relative to the benefit in the control arm. According to the Chinese Pharmacopoeia (2005 edition) [12], indications of XQLG include wind-cold-retained fluid pattern manifested as aversion to cold, fever, absence of sweating, wheezing, and cough with watery phlegm. In fact, our study demonstrates that if those patients with TCM symptoms can be separated out using latent class analysis, the effect of XQLG may be statistically significant when compared to placebo in this study.

Comparative latent class analysis is a statistical method to define symptom patterns for the purposes of distinguishing certain patient groups. This method was used in this study to analyze in greater depth the efficacy of the XQLG treatments. The determination of what treatment is best for what kinds of patients is a general objective of clinical research [51]. One major factor is the application of the LCA in an effort to determine which treatment is preferable for which kind of patients. Interestingly, results indicate that all the subjects can be classified into two distinct subsets according to the conditional probabilities of asthma participants found in the LCA. The symptom trend was similar in both classes, but there were clear differences. The subtype of 70 participants had a higher probability of reporting adherence to the six typical symptoms, including Qi vacuity, chest tightness, aversion to cold, clear, pale, or watery phlegm, torpid intake, and coughing. When this group was analyzed separately for the comparative efficacy of XQLG versus placebo add-on therapy, significantly increased levels of asthma control were found in the former group (*P* = 0.0329) [9]. The implications of this finding is that one may be able to use TCM symptom pattern recognition to study and subsequently identify in clinical practice those patients who may respond optimally to a specific herbal treatment.

There were no serious adverse events and side effects were insignificant, in line with other similar studies [13]. XQLG intervention appears to be a safe mode of therapy in our patient study group. However, in the United States, XQLD is not available from a pharmacy or via a doctor's prescription because this formula includes an illegal herb ephedra [52]. New evidence indicates that rational use can avoid the toxicity of ephedra [52]. It is important to note that ephedra must always be prescribed with care and the safe use of a combination of western medical remedies with herbal therapy should result from the evaluation of drug-herb interactions.

We strictly defined TCM indications in inclusion criteria. All subjects were characterized as TCM cold pattern by using the strict predefined criteria. However, we almost certainly underestimated pattern classification accuracy by using current criteria. If other TCM classifications of asthma are included, the results may be different [53, 54].

This study has other limitations. First, our subtype population was relatively small (class 1 = 100; class 2 = 70). Second, the use of multiple single-item symptom measures raises questions regarding reliability and validity. In the design of clinical trials on herbal remedies, a selection of only a few of the multitude of symptoms that asthma patients experience are evaluated. In this study, “asthma of cold pattern” according to current criteria of diagnosis and therapeutic effect of disease and syndromes and the diagnosis mild-to-moderate asthma might not suffice.

Despite these limitations, this study makes an important contribution to impact assessment of symptoms on the treatment efficacy in asthma patients. As we look toward future generations of TCM asthma trials, the concept of individualized therapy based on predictive symptom markers to better select patients who would benefit from a specific intervention is likely to be integral. A recent perspective on considering inclusion and exclusion criteria for randomized controlled trials (RCTs) may integrate genotype through characteristic symptoms [55]. It shows how meaningful stratification based on symptomatology may be, and how careful selection of patients for targeted therapies may be of utmost importance for developing future treatment strategies.

Further assessment is warranted of the role of XQLG in adult asthmatic patients with six TCM typical symptoms. If reproducible, these results provide support for designing interventions aimed at more definite symptoms in the millions of patients currently treated for asthma.

## 6. Conclusions

The addition of XQLG to existing treatment using combination inhaled corticosteroid/long acting beta-agonist (LABA) therapy did not demonstrate a statistically significant improvement over placebo add-on therapy. However, latent class analysis of Chinese medicine symptom patterns was able to define a subset of patients who would respond to XQLG add-on therapy significantly better than placebo. XQLG may therefore be effective in the treatment of a subgroup of asthma patients and additional studies should be conducted to investigate efficacy and safety of this herbal medication as first line therapy for asthma. This study also illustrates the importance of elucidating ways of defining selective patient populations with favorable response to specific treatment modalities.

## Figures and Tables

**Table 1:** Components of Xiaoqinglong granule (with sugar, 1000 g)* and their chinese medicine treatment functions.

Pi yin name	Latin name	Part used	Weight	Chinese medicine treatment functions
Ma Huang	Ephedra sinica Stapf.	Stems	154 g	Ma Huang resolves the exterior and diffuses the lungs, stops coughing, and levels panting.
Gui Zhi	Cinnamomum cassia Presl.	Twigs and branches	154 g	Gui Zhi resolves the exterior and scatters cold. When combined with Ma Huang, Gui Zhi strengthens Ma Huang's function of promoting diaphoresis.
Bai Shao	Paeonia lactiflora Pall.	Roots	154 g	Bai Shao is used to harmonize the blood to regulate qi.
Gan Jiang	Zingiber officinale Rosc.	Rhizomes	154 g	Gan Jian scatter cold, dispel dampness.
Xi Xin	Asarum heterotropoides Fr. Schm.	Whole	77 g	Xi Xin prevents wind cold.
Zhi Gan Cao	Glycyrrhiza uralensis Fisch.	Roots	154 g	Mix-fried Gan Cao harmonizes the other medicinal in this formula.
Fa Ban Xia	Pinellia ternate (Thunb.) Breit.	Rhizomes	231 g	Ban Xia transforms phlegm and down bears the qi.
Wu Wei Zi	Schisandra chinensis (Turcz.) Baill.	Fruits	154 g	Wu Wei Zi engenders fluids, constrains the lung qi, and helps stabilize panting.

*Chinese Pharmacopoeia (2005) [12].

**Table 2:** Two latent class models of asthma participants and relative symptoms response probabilities.

Item types (symptom)	Rule 1 Class 1 subset (*n* = 100)	Rule 2 Class 2 subset (*n* = 70)
Qi vacuity	**0.8903** ^†^	**1.0000**
Chest tightness	**0.7735**	**0.9672**
Aversion to cold	**0.6385**	**0.7454**
Clear, pale, or watery phlegm	0.3927	**0.9984**
Torpid intake	0.3554	**0.6935**
Coughing	**0.8604**	**1.0000**
Fever	0.0000	0.0287
No thirst	**0.7481**	**0.4588**

Latent class prevalence	0.5898	0.4102

*Note.* The model shows the different response patterns of the two rules on different item types. Rule  1; Rule  2.

^†^Item-response probabilities > .5 in bold to facilitate interpretation. Item-response probabilities corresponding to a yes response; Qi vacuity: shortness of breath; chest tightness: oppression in the chest, feeling of oppression in the chest; aversion to cold: sensation of cold which cannot be relieved by warmth, also known as chills; clear, pale, or watery phlegm; torpid intake: loss of appetite with no desire for food with decreased intake, the same as poor appetite; coughing: (1) the expelling of air from the lungs suddenly with an explosive noise or expectoration of sputum; (2) any disease mainly manifested by cough; fever: elevation of the body temperature above the normal or subjective feeling of feverishness; no thirst: no feeling of dryness of the mouth with a desire to drink.

**Table 3:** Subject demographics and baseline characteristics (ITT population).

	XQLG add-on group (*n* = 84)	Placebo add-on group (*n* = 86)
Age, year, mean (SD)	44.13 (12.42)	44.20 (11.92)
Sex, *n* male (%)	34 (40.48)	46 (53.49)
BMI, kg/m^2^, mean (SD)	22.66 (3.22)	22.72 (2.83)
GATPC		
II	45 (53.57)	44 (51.16)
III	39 (46.43)	42 (48.84)
Duration of disease, year, mean (minimum, maximum)	10.83 (0.00,52.00)	11.24 (0.00,54.00)
FEV_1_ (%), mean (SD)	75.60 (25.91)	72.45 (22.61)
PEF, L/min, mean (SD)	282. 71 (135.84)	282.31 (20.92)
ACT, mean score (SD)	16.86 (3.67)	16.94 (3.57)

Definition of abbreviations: GATPC: guideline of asthma treatment and prevention in China; ACT: asthma control test; ITT: intent-to-treat.

**Table 4:** Assessment of Level of asthma control according to GINA Guidelines.

Visit	Levels of asthma control	XQLG add-on group (*n*, %)	Placebo add-on group (*n*, %)	*P* value
0 day	Uncontrolled	76 (90.48)	80 (93.02)	0.5470
	Partly controlled	8 (9.52)	6 (6.98)	
	Controlled	0 (0.00)	0 (0.00)	

	Total	84	86	

90th day	Uncontrolled	5 (6.58)	9 (11.25)	0.8883
	Partly controlled	41 (53.95)	38 (47.50)	
	Controlled	30 (39.47)	33 (41.25)	

	Total	76	80	

**Table 5:** Asthma control test (ACT) score and asthma symptoms score in trial visit.

	Day	Difference in mean change Group A versus Group B (95% CI)	*P* value
ACT Score	7	0.26 (−1.31 to 1.82)	0.5914
	30	0.20 (−1.64 to 2.04)	0.7166
	60	0.65 (−1.19 to 2.49)	0.2485
	90	0.35 (−1.49 to 2.19)	0.5334

Asthma symptoms	7	−1.09 (−3.42 to 1.24)	0.1255
	30	−0.68 (−3.39 to 2.02)	0.4083
	60	−1.42 (−4.1 to 1.25)	0.0823
	90	−1.13 (−3.83 to 1.58)	0.1728

**Table 6:** Subject demographics and baseline characteristics of class 1 and 2 subset (*n* = 170).

	Class 1 subset (*n* = 100)	Class 2 subset (*n* = 70)	*P* value
Age, year, mean (SD)	43.67 ± 12.61	44.87 ± 11.46	0.5462
Sex, *n* male (%)	51 (55.00)	29 (41.43)	0.2199
BMI, kg/m^2^, mean (SD)	22.86 ± 3.39	22.46 ± 2.40	0.4990
GATPC, mean score (SD)			
II	53 (53.00)	36 (51.43)	0.8405
III	47 (47.00)	34 (48.57)	
Duration of disease, year, mean (SD)	12.38 ± 13.74	9.13 ± 10.52	0.2448
FEV_1_ (%), mean (SD)	72.76 ± 26.52	75.84 ± 20.73	0.7313
PEF, L/min, mean (SD)	287.6 ± 140.07	275.3 ± 117.32	0.4194

**Table 7:** Assessment of levels of asthma control according to GINA guidelines in class 1 and class 2 subset of participants.

	0 day			Mean score	*P* value	90th day			Mean score	*P* value
	Uncontrolled	Partly controlled	Controlled			Uncontrolled	Partly controlled
Class 1										
XQLG add-on group	47	5	0	50.80	0.8292	6	30	16	46.67	0.1291
Placebo add-on group	44	4	0	50.16		5	20	23	54.64	

Class 2										
XQLG add-on group	29	3	0	36.28	0.5173	2	15	15	40.65	0.0329
Placebo add-on group	36	2	0	34.84		8	20	10	31.15	

## Abbreviations

ACT: Asthma control test
AE: Adverse event
CAM: Complementary or alternative medicine
CHM: Chinese herbal medicine
GATPC: The guideline of asthma treatment and prevention in China
H1R: Histamine H(1) receptor
HDC: Histidine decarboxylase
ITT: Intent-to treat
IVR: Interactive voice response
IWR: Interactive web response
LCA: Latent class analysis
RCT: Randomized controlled trial
SAE: Serious adverse event
SCRT: So-cheong-ryong-tang
SR: Systematic review
SST: Sho-seiryu-to
TDI: Toluene-2,4-diisocyanate
XQLD: Xiaoqinglong decoction
XQLG: Xiaoqinglong granules.
